# Coincidence detection and integration behavior in spiking neural networks

**DOI:** 10.1007/s11571-023-10038-0

**Published:** 2023-12-13

**Authors:** Andreas Stoll, Andreas Maier, Patrick Krauss, Richard Gerum, Achim Schilling

**Affiliations:** 1 Pattern Recognition Lab, University Erlangen-Nürnberg, Erlangen, Germany; 2 Neuroscience Lab, University Hospital Erlangen, Erlangen, Germany; 3 Department of Physics and Astronomy, York University, Toronto, Canada

**Keywords:** Computational modeling, Neural networks, Artificial intelligence, Leaky-integrate-and-fire neuron, Coincidence detection

## Abstract

Recently, the interest in spiking neural networks (SNNs) remarkably increased, as up to now some key advances of biological neural networks are still out of reach. Thus, the energy efficiency and the ability to dynamically react and adapt to input stimuli as observed in biological neurons is still difficult to achieve. One neuron model commonly used in SNNs is the leaky-integrate-and-fire (LIF) neuron. LIF neurons already show interesting dynamics and can be run in two operation modes: coincidence detectors for low and integrators for high membrane decay times, respectively. However, the emergence of these modes in SNNs and the consequence on network performance and information processing ability is still elusive. In this study, we examine the effect of different decay times in SNNs trained with a surrogate-gradient-based approach. We propose two measures that allow to determine the operation mode of LIF neurons: the number of contributing input spikes and the effective integration interval. We show that coincidence detection is characterized by a low number of input spikes as well as short integration intervals, whereas integration behavior is related to many input spikes over long integration intervals. We find the two measures to linearly correlate via a correlation factor that depends on the decay time. Thus, the correlation factor as function of the decay time shows a powerlaw behavior, which could be an intrinsic property of LIF networks. We argue that our work could be a starting point to further explore the operation modes in SNNs to boost efficiency and biological plausibility.

**Supplementary Information:**

The online version of this article (10.1007/s11571-023-10038-0) contains supplementary material, which is available to authorized users.

## Introduction

Biological neural networks and especially the human brain achieve astonishing performance in dynamical information processing and energy efficiency. Thus, the human brain does not consume significantly more power than a 20 W light-bulb (Furber 2012), whereas huge matrix processor units used for machine learning approaches consume far more energy (Wang et al. 2020). How is it possible that general intelligence emerges in the human brain, although there exist significant biological constraints? On the one hand, the answer to this question has the potential to significantly boost artificial intelligence (AI) research by implementing the underlying biological principles in artificial neural networks [neuroscience inspired AI, (Hassabis et al. 2017), e.g. (Yang et al. 2021; Schilling et al. 2022)]. On the other hand, better artificial neural networks could help to understand how the brain works, as these networks could serve as a model system which can be analyzed with much more detail compared to their biological counterpart [cognitive computational neuroscience, (Kriegeskorte and Douglas 2018), see also (Gerum et al. 2020; Schilling et al. 2021a; Stoewer et al. 2023a, b; Surendra et al. 2023; Metzner et al. 2023; Schilling et al. 2022)].

Consequently, the interest in neuromorphic computing and especially spiking neural networks (SNNs) increased in recent years, as they offer a promising approach to bridge the gap between the performance achieved by current deep learning methods and the energy efficiency of biological neural networks (Eshraghian et al. 2021; Yamazaki et al. 2022; Xiao et al. 2022; Gerum et al. 2023).

The behaviour of biological neurons has first been mathematically described by Hodgkin and Huxley (1952). Even though the Hodgkin–Huxley model accurately describes the underlying experimental observations, it is computationally complex and therefore often simplified. One simplification is for example the Fitzhugh–Nagumo neuron model, which uses a reduced set of differential equations (FitzHugh 1961; Izhikevich and FitzHugh 2006; Nagumo et al. 1962). However, to simulate large networks based on these neuron models is computationally expensive and unfeasible. Therefore, biological neurons are commonly approximated with phenomenological spiking neuron models (Gerstner and Kistler 2002). Spiking neurons produce identical action potentials (spikes) when certain threshold criteria are met for their internal states (Kandel et al. 2000). As a result, these neurons transmit information via an energy-efficient communication scheme that is binary and sparse.

One prevalent spiking neuron model is the leaky-integrate-and-fire (LIF) neuron. It is computationally efficient and therefore used in many spiking neural network studies (Yamazaki et al. 2022).

SNNs provide a more biologically-inspired approach to artificial neural networks compared to standard deep neural networks used for pattern recognition. However, training SNNs remains challenging and is an active field of research (Xiao et al. 2022; Alonso et al. 2022; Apolinario and Roy 2023; Gerum and Schilling 2021; Gerum et al. 2023). Even though simple models like the LIF neuron provide a wide range of parameters that influence the dynamics of information processing, many recently proposed training methods, e.g. Xiao et al. (2022) and Apolinario and Roy (2023), still only optimize the synaptic weights. This is likely due to a lack of understanding the effects of different parameters on the spiking dynamics of SNNs.

Recently though, multiple publications set a starting point to evaluate how SNNs behave under different conditions and parameter combinations. More so, a special focus was set on the temporal parameters (resp. time constants) of LIF neurons. For example, Perez-Nieves et al. (2021) show that SNNs perform best, when there is a certain heterogeneity in the time constants of LIF neurons and report a special benefit for tasks with a lot of temporal structure in the data. Further studies carried out similar experiments, but the authors set their focus on running SNNs more efficiently on neuromorphic hardware (Fang et al. 2021; Quax et al. 2020; Yin et al. 2020). In Gerum and Schilling (2021), the authors proposed that a single LIF neuron can be run in two operation modes: a LIF neuron with a short membrane decay time can be regarded as coincidence detector whereas a LIF neuron with a long membrane decay time acts as integrator neuron. However, it is still unclear whether these operations modes actually arise, and thus, whether they can be precisely tuned in populations of neurons (resp. SNNs).

Coincidence detection in particular seems to be a desirable operation mode, as it was observed in many different sensory modalities and cognitive processes by multiple studies of biological neural networks. It is known to be involved in e.g. memory formation (Bender et al. 2006; Fino et al. 2010) and decoding motor input or sensory stimuli (Xu et al. 2012; Roome and Kuhn 2020). Coincidence detection was also found to be involved in the auditory (Franken et al. 2015) and visual system (Ran et al. 2020) of mammals, respectively.

Detecting a coincidence refers to the process of extracting information from activity across different neurons which occurs within a short period of time. However, there are differences both in what kind of coincidence a system is trying to detect as well as the mechanisms of detecting such. In this study, coincidence detection refers to a postsynaptic neuron being prone to pre-synaptic activity that arrives over a short period of time.

A biological mechanism for detecting this kind of coincidence has been reported to exist in e.g. the auditory system of the Mongolian gerbil (Franken et al. 2015) and is crucial for sound localization. Intrinsic conductances of neurons in the medial superior olive—a brainstem nucleus of the auditory pathway—that interact with preceding synaptic activity are reported to generate an internal phase delay as part of the coincidence detection process. Both the recent input activity as well as low-voltage-activated Kv1 potassium channels alter the postsynaptic neuron’s membrane potential which enables fine tuned responses to different temporal input patterns. This biological mechanism is involved in spatial hearing, as the coincidence detection allows to resolve the small time difference of a sound arriving at the two ears. Further studies on the auditory system proposed that coincidence detection is also important to generate neural networks, which are able to calculate auto-correlations of complex signals. Thus, Krauss et al. (2016, 2018), Schilling et al. (2021b), Schilling and Krauss (2022) and Schilling et al. (2023) propose that these auto-correlations of auditory signals are used by the auditory system to enhance sensory processing and to compensate the effects of hearing loss.

Despite a central role of coincidence detection in the auditory system, it is important in various other modalities and brain regions as well. Coincidence detection also plays a role in e.g. cortical integration of sensory and motor input (Xu et al. 2012), sub-cortical processing of visual stimuli (Ran et al. 2020) and information processing in the cerebellum (Roome and Kuhn 2020).

Regardless of numerous evidence of this operation mode to exist in biological neural networks, its potential is yet to bet tapped into by a majority of SNN studies. In this study, we therefore explore the connection between the membrane time constant and the proposed LIF operation modes in SNNs that are trained on four commonly used image classification datasets: MNIST, EMNIST/Letters, Fashion-MNIST and CIFAR-10. We propose two measures, that allow to determine a neuron’s operation mode with respect to the other neurons in a spiking neural network. Thus, the proposed measures can be used to better understand the contribution of single neurons to the dynamics of entire populations of neurons. Besides supporting the explainability of SNNs, we also demonstrate a clear correlation between the membrane decay time (inverse leak term) and the neuron’s spiking dynamics in SNNs optimized with a supervised surrogate-gradient-based training method. We find, that the coincidence detection mechanisms observed in biology can be reproduced in networks of LIF neurons in a simplified manner. This makes tuning the operation modes (resp. membrane decay times) an interesting approach to more biological plausibility in machine learning.

## Methods

### Computational resources

All simulations were performed on standard Desktop PC hardware. The experiments were run on a modified version of the tf_spiking Python package (Gerum 2020b) which is the backbone of our machine learning approaches and based on Keras (Chollet et al. 2015) and TensorFlow (Abadi et al. 2015). For further evaluations, we used NumPy (Harris et al. 2020) and Pandas (The pandas development team 2020). Thus, all visualizations were created with Matplotlib (Hunter 2007) and Pylustrator (Gerum 2020a). The experiments were conducted with a five-fold cross-validation on four different image classification datasets, namely the MNIST database of handwritten digits (Deng 2012), EMNIST/Letters (Cohen et al. 2017), Fashion-MNIST (Xiao et al. 2017) and CIFAR-10 (Krizhevsky et al. 2009). The image pixels are converted into spike trains using Poisson encoding.

Our fully connected network has one hidden layer of 128 LIF neurons and is supervisedly trained using the surrogate-gradient approach proposed in (Gerum and Schilling 2021). The membrane decay times (resp. leak terms) are initialized either with identical values for all neurons in the hidden layer ("constant") or with 32 bins of four neurons each ("binned uniform"). The neurons of a bin are initialized with the same membrane decay time.

### Deep learning with leaky-integrate-and-fire neurons

**Fig. 1:**
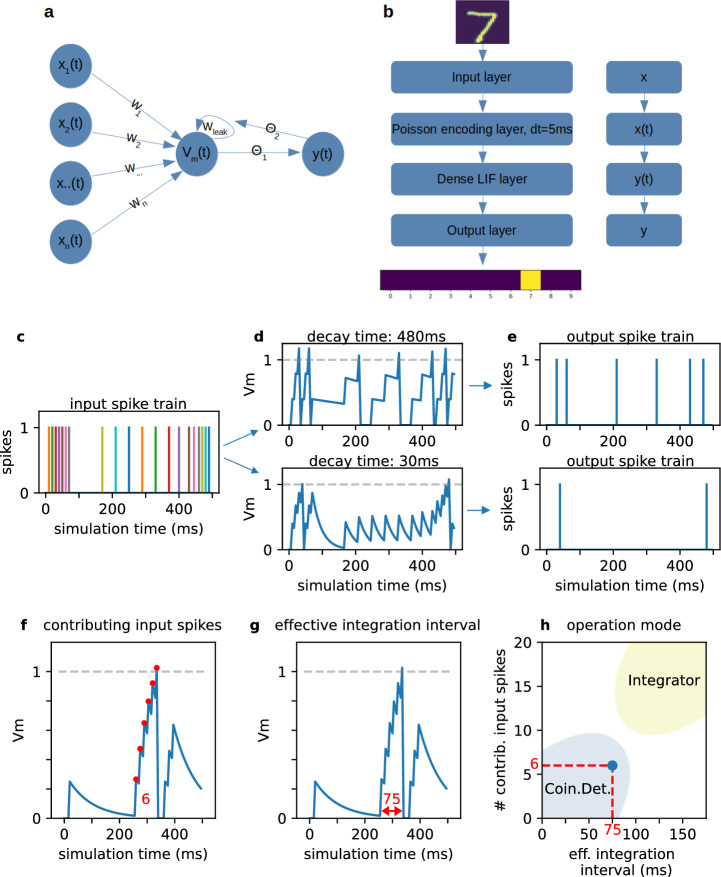
**a**: The leaky-integrate-and-fire neuron describes the relationship between input currents $x_{i}(t)$ and the output current *y*(*t*). **b**: The feed-forward network architecture used in our study with an exemplary MNIST input image and the resulting classification. Whether the signal is or isn’t time-dependent is indicated on the right. Given the input spike train (**c**), a neuron’s membrane potential behaves differently according to its decay time (**d**). If it is high (480 ms), $V_\text{m}$ stays above its resting state for some time and can be integrated over multiple time steps. If it is low (30 ms), the spike threshold is only surpassed when either strong or lots of input activity rapidly stimulates the neuron. The respective output spike trains are visualized in **e**. The number of contributing input spikes (**f**) and the effective integration interval (**g**) are determined by backtracking input spikes in $V_\text{m}$ after an output spike was elicited. During this process, weight as well as membrane decay effects are being considered. By combining these measures (**h**), we can determine a neuron’s operation mode in the context of the total simulation time of the network

For our experiments, we build a feed-forward spiking neural network based on leaky-integrate-and-fire (LIF) neurons (Burkitt 2006) (see Fig. 1a). As shown in Gerum and Schilling (2021), LIF neurons can be mathematically described by the following equations:

1 $$\begin{aligned} V_{t_{n}} =&\text{ReLu}[ w_{\text{input}} \cdot x_{t_{n}} \nonumber \\&+ (1-w_{\text{leak}} \cdot \Delta t) \cdot V_{t_{n-1}} \cdot \Theta _{2}(V_\text{thresh} - V_{t_{n-1}}) ] \end{aligned}$$

2 $$\begin{aligned} y_{t_{n}} =&\Theta _{1}(V_{t_{n}} - V_\text{thresh}) \end{aligned}$$

3 $$\begin{aligned} t_{n} =&t_{n-1} + \Delta t \end{aligned}$$

$$V_{t_{n}}, V_\text{thresh}, x_{t_{n}} \in \mathbb {R}, w_\text{leak}, \Delta t, y_{t_{n}} \in \mathbb {R}^+, n \in \mathbb {N}$$

We simulate the neuron for $n = 1,..., N$ discrete time steps with a temporal resolution of $\Delta t =$ 5 ms. The internal state $V_{t_{n}}$ of the LIF neuron, also referred to as membrane potential, is computed for every time step $t_{n}$. It is the sum of the inputs at this time, $x_{t_{n}}$, weighted by trainable input weights $w_\text{input}$, and the state of the membrane potential of the previous timestep $V_{t_{n-1}}$, weighted by leakage term $w_\text{leak}$ that prevents long temporal correlations. As this study investigates the influence of the leakage term on the network dynamics, $w_\text{leak}$ is set to be non-trainable and therefore remains unchanged for all time steps. With the Heaviside step function $\Theta _{i}$, we can model the neuron to release a spike ($\Theta _{1}$ in (2)) and to reset the membrane potential ($\Theta _{2}$ in (1)) to its resting state, if $V_{t_{n}}$ surpasses the threshold $V_\text{thresh}$ at the respective time step. If the threshold is not surpassed, $V_{t_{n}}$ is multiplied with $w_\text{leak}$ and fed to the inner state via a recurrent connection. The output of a LIF neuron, $y_{t_{n}}$, is 0 if no spike occurs at $t_{n}$ and 1 otherwise. Without loss of generality, $V_\text{thresh}$ is set to 1. As we work with spike trains as input signals, both $x_{t_{n}}$ as well as $y_{t_{n}}$ implicitly are binary, i.e. $\{0, 1\}$, and independent of $\Delta t$. We do not allow negative values for the inner state of the LIF neurons [cf. (2), (Gerum and Schilling 2021)].

For training the SNN, we work with the surrogate gradient-based backpropagation through time approach proposed in Gerum and Schilling (2021). With this learning paradigm, supervised training by minimizing a loss function is possible and classification tasks can be solved. The loss function, in our case the mean squared error loss, is minimized by using a gradient descent algorithm and a step size (learning rate). For the optimization we use the Adam stochastic gradient descent method (Kingma and Ba 2017). Thus, a weight update can be calculated via the chain rule the same way as for multi-layer-perceptron-based artificial neural networks.

As we work with image datasets but the LIF neurons have a time dimension, all image pixels are encoded as spike trains. In this study, we use Poisson rate coding (Zenke and Vogels 2021; Pfeiffer and Pfeil 2018; Lee et al. 2016), where every pixel value is translated to a probability of the neuron to spike in each time step. After the encoding, the inputs are passed to a dense layer consisting of 128 LIF neurons. In order to get a final classification score consistent with the ground truth labels, the output layer sums up the incoming spikes from the hidden layer and maps it to the respective class. We simulate the network for a duration of 500 ms with a temporal resolution of 5 ms, resulting in 100 discrete time steps. An overview of the network architecture is visualized in Fig. 1b.

### Tuning the spiking behavior of LIF neurons via their decay times

The decay time is inversely proportional to the leak term ($t_\text{decay} = 1 / w_\text{leak}$) and influences both training dynamics and spiking behavior of the LIF neurons. For a high decay time (resp. low leakage), a LIF neuron simply sums up the input stimuli over time with little decay of the membrane potential and therefore operates as integrator. On the contrary, if the neuron’s decay time is low (resp. high leakage), it can only release output spikes for inputs with small time differences. In such a case, the LIF unit operates as coincidence detector. In Fig. 1c–e, we visualized the membrane potential of a single LIF neuron with a high (low) decay time and the resulting output behavior given an identical synthetic input spike train, respectively.

We refer to these operation modes as integrator and coincidence detector, however, both terms are not correlated to a specific value for the decay time but rather describe the neuron’s tendency of operation in the context of the network’s simulation time. Neurons with intermediate decay times (resp. an intermediate operation mode) can of course exist and thus the proposed terms for the operation modes strongly depend on the context of the input stimuli.

**Fig. 2:**
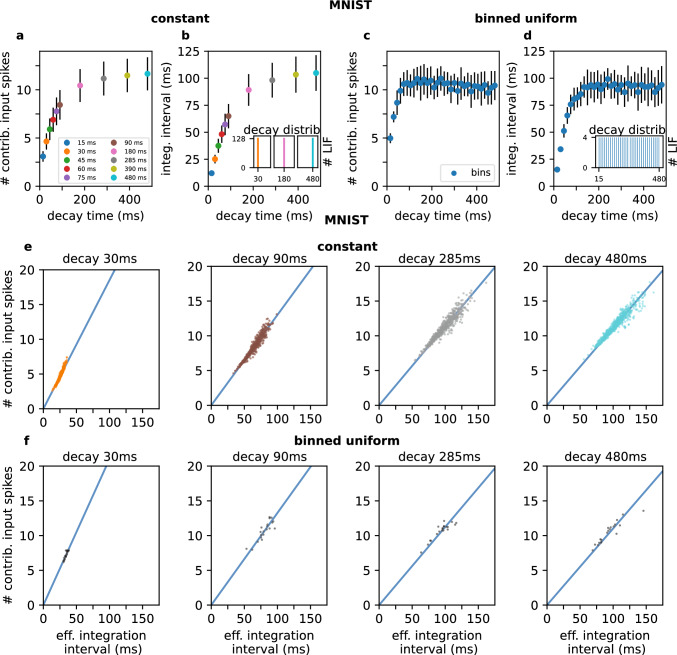
Both measures are influenced by the decay time. The two measures we introduced to determine the neuron’s operation mode are clearly influenced by the decay time in networks trained on MNIST. The results of the experiments using constant decay times are shown in **a** and **b**. Every point denotes an experiment with exclusively using the respective decay time. **c** and **d** show the results of the experiment using binned uniformly distributed decay times. Here, every point denotes one bin of neurons with the respective decay time. The average numbers of contributing input spikes are visualized in plots **a** and **c**; the effective integration intervals are plotted in **b** and **d**. The decay time impacts both measures similarly in experiments with constant as well as binned uniform initialization.When visualizing the number of contributing input spikes and the respective effective integration interval w.r.t. a specific decay time as scatter plot, a linearly-shaped distribution forms which gets steeper and more compact for lower decay times. This trend was observed to be identical in constant (**e**) and binned uniform (**f**) experiments. These results suggest that the decay time determines a neuron’s operational range rather than an exact operation mode. The colored lines show linear fits through the respective distributions

As our learning rule depends on the membrane potential, the decay time also affects the training behavior of the neuron. An integrator neuron can memorize its inputs over a long duration, so the error gradient also has to be backpropagated over a longer duration than it is the case for coincidence detector neurons. Tuning the decay time therefore allows modeling populations of neurons with different spiking behavior and memorization properties. For our experiments we consider decay times in the range [15, 480] ms. This interval roughly covers the simulation time of the neurons (500 ms) and excludes possible sampling artifacts given our choice of temporal resolution (5 ms). Thus, we can split this range into 32 equidistant bins with 15ms time difference. Via this approach we can distribute the decay times uniformly throughout the network with four neurons sharing a respective decay time. We refer to this initialization scheme as "binned uniform" (visualized in the inset in Fig. 2d).

## Results

### Coincidence detection and integration behavior in feed-forward spiking networks

In the following, we analyze how coincidence detector and integrator neurons (Gerum and Schilling 2021) perform and behave in a feed forward neural network trained with a surrogate gradient algorithm. Thus, we study the effect of different decay times on the spiking behavior in networks trained on four common datasets (MNIST, EMNIST/Letters, Fashion-MNIST or CIFAR-10 dataset). We investigate networks with constant decay times (i.e. it is equal for all LIF units of the network), and with binned uniform decay times, (i.e. it is uniformly distributed with an equal amount of neurons sharing a respective decay time). Thus, the decay time is identical for all time steps and is not a trainable parameter. For better readability, we only report the results based on MNIST in this Section; the visualizations for the other datasets can be found in Suppl. Fig. 1.

The operation mode of the neurons is quantified by the number of input spikes effectively contributing to the generation of an output spike (see Fig. 1f). A low number of contributing input spikes suggests that either the weights of these input spikes were very high, or that an input spike volley (several spikes coming from arbitrary input neurons with small inter-spike intervals) was present. As we are particularly interested in detecting coincidences, we additionally measure the average time interval in which the input spikes stimulate the LIF unit and cause it to spike. We call this the effective integration interval (see Fig. 1g).

To calculate this measure, we start with the time of the output spike and trace the membrane potential back to the first input spike that actively contributes to the generation of this output spike. During this backtracking, weight effects and membrane decay effects are being taken into account. A long effective integration interval (w.r.t. the network’s simulation time) is present in integrator neurons due to little decay of the membrane potential. We expect that coincidence detector neurons have shorter integration intervals compared to integrator neurons and require less input spikes.

We compute both measures for every output spike of every neuron in the hidden layer and use them to determine the operation modes of the neurons: If both measures (effective integration interval and contributing input spikes) are low the neuron operates as coincidence detector. If both measures are high the neuron operates as integrator as indicated in Fig. 1h.

Prior to analyzing whether a low number of contributing input spikes correlates with a short effective integration interval in networks trained on real data, we investigated the decay time’s influence on both measures. Low decay times correspond to low measure scores in experiments using constant as well as binned uniformly distributed decay times, as reported in Fig. 2a–d.

Visualizing both measures in the "operation mode" scatter plot (introduced in Fig. 1h), we find that a low (high) number of contributing input spikes correlates with a short (long) effective integration interval (see Fig. 2e, f). However, we find the decay time to not determine the exact behavior of the neuron but instead defines a range of operation. This range gets smaller for low and saturates for high decay times, respectively. These results imply, that we can in fact influence the operation mode of a LIF neuron via its decay time (resp. leak term). Furthermore, we see that in a population of neurons trained on real data, integration and coincidence detection behavior emerge depending on the decay time. The networks did not simply adjust the weights to counter the effect of the decay time, but instead worked with neurons operating on different time scales.

**Fig. 3:**
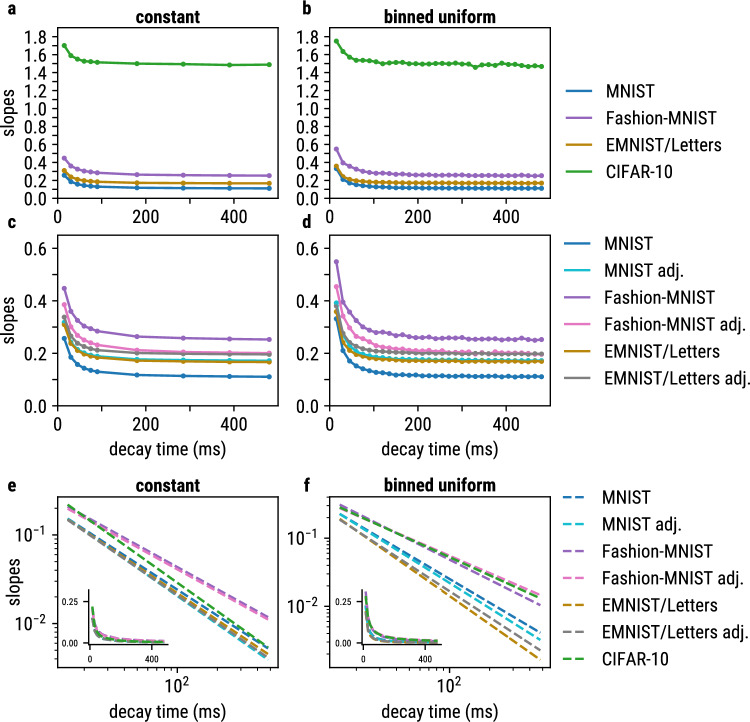
The slopes of the line fits in the scatter plots correlate with the decay time for both constant (**a**) and binned uniform (**b**) initialization. The impacts of different decay times are similar between the different datasets, however, the brightness of the input data produces an offset along the y-axis. After adjusting the average brightness of MNIST, EMNIST/Letters and Fashion-MNIST to approximately match, the curves are close in constant (**c**) and binned uniform (**d**) experiments. As they are not entirely aligned, the structure of the data also seems to effect the slopes. **e** and **f**: In order to compare the slopes between the different datasets, we subtracted the y-offset and fitted powerlaws to the curves from **a** and **b**, respectively. The powerlaw fits are presented in double logarithmic scale. For adjusted and non-adjusted data, the curves are closely aligned. In contrast, the powerlaw fits of different datasets only approximately match, suggesting an influence of the structure within the data. Remarkably, we observe the curves of MNIST and EMNIST/Letters to closely match in the binned uniform experiments, as is also the case for Fashion-MNIST and CIFAR-10, respectively. This suggests, that the distribution of the pixel intensities shapes the slope of the curve

The slopes of the drawn measurement values shown in Fig. 2e and f become steeper for lower decay times when training on EMNIST/Letters, Fashion-MNIST and CIFAR-10 data, respectively (see Suppl.Fig. 1 B). We therefore fitted lines through every distribution and the coordinate origin and computed their slopes. When plotting the slopes of these line fits over their respective decay time, we find similar curves for constant and binned uniform experiments and for all four datasets, as shown in Fig. 3a and 3b.

An in-depth analysis of these slopes as a function of the decay times (Fig. 3) indicates that the slopes of the curves are shifted along the y-axis depending on the dataset.

We found that the offset along the y-axis is influenced by image brightness. We therefore adjusted the average brightness of MNIST, EMNIST/Letters and Fashion-MNIST images to approximately match (brightness difference < 0.045 in [0, 255] color space, see Fig. 3c, d) However, we also see that curves from brightness-adjusted MNIST and unmodified EMNIST/Letters are similar, despite an average brightness difference of approx. 10 in [0, 255] color space. This suggests that not only image brightness, but also the structure of the data influences the slopes.

Additionally, when removing the y-offsets and fitting powerlaws to the resulting curves, we can compare the slopes for neural networks trained on the different datasets. The powerlaw fits are presented in double logarithmic scale in Fig. 3e, f. The curves of brightness-adjusted and non-adjusted data are very similar indicating that image brightness influences the y-offset but has little impact on the shape of the curve. In general, the different powerlaw fits are not perfectly aligned, potentially due to an influence of the structure of the data. Furthermore, we observed that the fit functions for networks trained on MNIST and EMNIST/Letters datasets are similar for the binned uniform experiments. Also the fit functions of Fashion-MNIST and CIFAR-10 experiments are similar. We therefore assume that the distribution of the pixel intensities shapes the slope of the curve, as MNIST and EMNIST/Letters are almost binary in intensity, whereas Fashion-MNIST and CIFAR-10 more extensively use the offered value range of the brightness scale.

### Impact of decay times on model accuracy

**Fig. 4:**
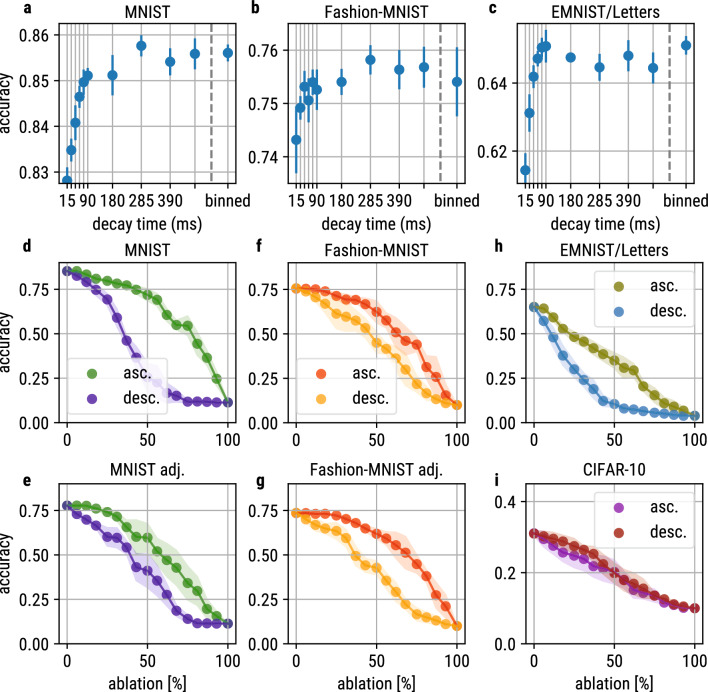
Accuracy and cumulative decay-based ablation. The accuracy scores of networks trained on MNIST (**a**), EMNIST/Letters (**b**) and Fashion-MNIST (**c**) are visualized w.r.t. the decay time of experiments using constant initialization. In the binned uniform experiment, the decay times were distributed equidistantly over 32 bins in the range [15, 480] ms. Every dot shows the five-fold cross-validated mean accuracy and the respective standard deviation. The accuracy is similar between most decay times within every dataset, however a tendency to decrease for low decay times can be noticed. The experiments using binned uniform initialization achieve approx. equal accuracy to the best models using constant decay times. The impact of different decay times on classification accuracy can be determined by cumulatively ablating neurons from the binned uniform experiments w.r.t. their decay times. We start with ablating coincidence detectors (ascending decay time) or integrators (descending decay time) first, respectively. The results for all datasets are visualized in **d, f, h, i**. For MNIST, EMNIST/Letters and Fashion-MNIST, ablating neurons with high decay times first, i.e. integrators, (desc. decay time) more rapidly results in a major loss of accuracy compared to ablating coincidence detectors first (asc. decay time). Comparing the models trained on MNIST (**d**) and Fashion-MNIST (**f**) to their adjusted versions (**e** and **g**), respectively, the image brightness seems to influence the importance of the different operation modes. A higher brightness in MNIST images decreases the difference of the ascending and descending ablation curves, while reducing the brightness in Fashion-MNIST images increases the difference of the curves

Different decay times lead to changes in spiking dynamics. In the following, we show the influence of the different decay times on classification accuracy. Therefore, we evaluated the five-fold cross-validated mean accuracy, macro f1-score and area under the receiver operating characteristic (AUC) for all experimental conditions. In Fig. 4a–c, the accuracies are reported for MNIST, Fashion-MNIST and EMNIST/Letters (detailed performance overview of accuracy, macro f1 and AUC scores of all datasets see Suppl. Figure 1 C).

The binned uniform distributions lead to similar performances as the best models with constant decay time.

In a next step, we investigated the influence of the decay time on overall classification accuracy by cumulatively ablating neurons either starting with coincidence detectors (starting ablation at low decay times) or integrator neurons (starting ablation at high decay times), respectively. The results for all datasets are reported in Fig. 4d-i. Deleting integrator neurons first leads to an instant accuracy drop for MNIST (4d), Fashion-MNIST (4f) and EMNIST/Letters (4h) datasets. For CIFAR-10 (4i) no preference of operation mode can be detected.

Comparing the ablation curves of MNIST (4d) and brightness-adjusted MNIST (4e), we find that increasing the image brightness leads to a smaller difference between ascending (green) and descending (blue) ablation curves. Consistently, a greater difference is observed when lowering the image brightness of Fashion-MNIST (see 4f, g). This therefore suggests, that the performance difference between integrator and coincidence detector neurons is linked to image brightness.

In summary, integrator neurons seem to be more important for classification performance. However, this result has to be discussed due to the observed dependence on image brightness (resp. spike rate given the Poisson spike encoding).

## Discussion

### Summary

In the present study, we investigated whether the previously proposed operation modes of single LIF neurons do emerge in spiking neural networks that are trained on real data. We thus studied the influence of tuning the membrane decay time [i.e. membrane time constant (Perez-Nieves et al. 2021) or inverse leak term] on the operation modes of LIF neurons. Furthermore, we analyzed the resulting effects on spiking dynamics and image classification accuracy of SNNs. We found that the proposed operation modes do emerge in SNNs and that they can be tuned via the membrane decay time: Neurons with low decay times operate as coincidence detectors, whereas neurons with high decay times operate as integrators.

We performed experiments with four image datasets (MNIST, EMNIST/Letters, Fashion-MNIST and CIFAR-10), which were transformed to spike sequences by Poisson encoding [Zenke and Vogels 2021; Pfeiffer and Pfeil 2018; Lee et al. 2016]. We deployed feed-forward SNNs with a single hidden layer and used the surrogate-gradient-based training method proposed by Gerum and Schilling (2021). In this study, we experimentally investigated the effect of different membrane decay times and considered two different ways of initializing them: constant decay times and uniformly distributed decay times, respectively.

In order to study the relationship between membrane decay times and operation modes of LIF neurons, we proposed two measures: the number of contributing input spikes and the effective integration interval.

We found the first measure, the number of contributing input spikes, to be low for coincidence detectors, whereas it was found to be high for integrator neurons. The second measure, the effective integration interval, was also found to be higher for integrator neurons.

We analyzed the distribution of the two measures across the SNNs with respect to particular membrane decay times and found the two measures to linearly correlate. Besides investigating the relationship between the measures and a neuron’s operation mode, we found that both measures are strongly influenced by the neuron’s membrane decay time (see Fig. 2a–d).

Therefore, we can conclude that the operation mode of a LIF neuron can be determined via the membrane decay time. A low decay time correlates to a low number of contributing input spikes and a short effective integration interval. This consequently makes a LIF neuron to operate as coincidence detector. High decay times correspond to high numbers of contributing input spikes as well as long effective integration intervals. This makes such neurons to operate as integrators. However, saturation effects for high decay times were visible in both measures. This suggests a non-linear relationship between the membrane decay time and a neuron’s respective operation mode.

Our experiments give strong evidence that LIF neurons can be precisely tuned towards detecting coincidences, whereas integration behavior is not accurately determined: our analyses show, that the effective integration interval and number of contributing input spikes are more strongly determined towards a precise range (i.e. clustered together) for low decay times (see Fig. 2e, f). We can therefore conclude that the membrane decay time defines a neuron’s operational range rather than an exact operation mode. This operational range gets smaller (resp. more precise) for lower decay times.

Additionally, we found that the correlation factor between the two measures as a function of the membrane decay time follows a powerlaw. Therefore, the decay time offers a different way of influencing the spiking dynamics compared to the synaptic weights: while the weights linearly influence the spiking behavior of a LIF layer, the membrane decay time influences the spiking dynamics in a non-linear way.

The powerlaw relation between the measures and the decay time was present in all experiments and all datasets and we therefore argue that it is an intrinsic property of LIF neurons. However, we found it to slightly differ between different datasets and it is still not completely clear to which proportion the powerlaw relation is influenced by the structure of input data. We observed the brightness of the input data to linearly shift the curve of the correlation factor but to have little influence on the slope of the curve (see Fig. 3c–f). The observed similarities of the fit functions between MNIST and EMNIST/Letters and those between Fashion-MNIST and CIFAR-10, respectively, thus suggest that the exact shape of the powerlaw is indeed influenced by the structure of input data, e.g. the distribution of the pixel intensities.

Besides showing the emergence of coincidence detectors and integrators in SNNs and analyzing the resulting effects on the spiking dynamics, we explored the impact of different operation modes on image classification accuracy. For that, we cumulatively ablated neurons according to their decay times. Neurons were ablated either in ascending or descending order, respectively (see Fig. 4d–i).

Ascending ablation refers to deleting coincidence detectors first, which forces the network to use neurons that operate as integrators.

Descending ablation refers to deleting integrator neurons first, which forces the network to use neurons that operate as coincidence detectors.

We found that ablating integrator neurons had a more severe effect on classification accuracy than ablating coincidence detectors, which indicates that integrator neurons are more important in our experiments.

Additionally, we found the differences between the ascending and descending ablation curve to be strongly dependent of image brightness. This is likely due to encoding the images via a Poisson process. A brighter pixel is represented with a higher probability of the respective input neuron to spike. Consequently, the spike rate of such neurons is higher compared to neurons that encode darker pixels. Therefore, when the image brightness decreases, the spiking activity in the SNNs gets more sparse and consequently, fewer spikes coincide. In order to achieve good classification performance in such a case, integrator neurons, i.e. long decay times, are required, as they are able to memorize the information carried by spikes over a longer duration.

### Limitations of the study

It has to be noted, however, that the Poisson process encodes all the information of an image pixel via the spike rate of the respective input neuron. As a result, detecting a coincidence in our experiments does not provide more/different but less information than simply integrating over an arbitrary amount spikes. Because of that, we found integrator neurons to be more important than coincidence detectors in our experiments in terms of classification performance.

Even though this limits the results of our ablation study, we can conclude that when working with spike rates, tuning the membrane decay times can be neglected and training the synaptic weights is sufficient in order to achieve good classification performance. However, Perez-Nieves et al. (2021) showed that tuning the time constants can result in improved network performance, when information is not only encoded in the spike rate but in the spike timing as well. We therefore argue that when working with such data, tuning the membrane decay times of LIF neurons should be taken into account. This can be achieved either by considering the membrane decay times as trainable parameters as proposed by Gerum (2020b), or alternatively, by considering the distribution of decay times as hyper-parameter as we did in this study.

We thus want to emphasize that the timings of spikes are important when working with data from neuromorphic sensors like dynamic vision sensors or artificial cochleas (Eshraghian et al. 2021). We will therefore shift our focus to working with neuromorphic sensor data in the future.

Also, we only considered small feed-forward SNNs in this study due to the computational complexity induced by training SNNs [see also (Perez-Nieves et al. 2021)]. This limits our experimental evidence, as more complex effects could potentially emerge in larger—or even recurrent—spiking neural networks. Still, our experimental setup is a reasonable choice as there currently is no viable alternative to training SNNs in time-stepped simulation frameworks when good performance needs to be achieved (Eshraghian et al. 2021).

### Discussion and future research directions

In summary, we could demonstrate the rich dynamics of LIF-based spiking neural networks trained with surrogate gradient descent and provide evidence for the validity of defining two operation modes of LIF neurons: the integrator and coincidence detector.

We show that the coincidence detection mechanisms that have been observed in biological neural networks by multiple studies can be reproduced in LIF neurons by tuning the membrane decay times.

A recent study already showed that heterogeneous time constants can improve the performance of LIF-based SNNs (Perez-Nieves et al. 2021). Much work was already spend on investigating the effect of weight matrix heterogeneity on network dynamics [e.g. (Krauss et al. 2019b; Yang et al. 2021; Krauss et al. 2019c, a)]. However, only recently the exact temporal dynamics have moved into the focus of AI research.

As the temporal dynamics of LIF neurons are influenced by multiple parameters (e.g. membrane time constant, spike rate adaptation, data encoding), our aim was to disentangle these parameters and to study the impact of different membrane decay times. With this study, we contribute towards better understanding the dynamics of SNNs by providing experimental evidence for the emergence of different neural operation modes and their dependence on the membrane decay time.

Because the timing of spikes is important when working with neuromorphic sensor data, we strongly encourage the neuromorphic community to consider tuning the operation mode of LIF neurons in future experiments and to consider the membrane decay time in new training methods.

### Concluding remarks

To the best of our knowledge this study is the first to investigate the integration and coincidence detection behavior of LIF neurons in spiking neural networks and we thus provide a valid contribution to decode the basis of heterogeneity as fundamental principle of brain dynamics and efficient information processing in SNNs.

As already suggested in Jonas and Kording (2017), the best way to understand a complex system like the brain or artificial neural networks, is to search for already known building blocks (i.e. integrator and coincidence detector). To put it in a nutshell, a mechanistic theory is necessary to make real progress in understanding cognition in biological and artificial neural networks (Jonas and Kording 2017; Schilling et al. 2023).

### Supplementary Information

Below is the link to the electronic supplementary material.

Supplementary file1 (PDF 1579 kb)

## Data Availability

The current study was conducted using publicly available datasets.

## Code Availability

The code generated during the current study is available in the GitHub repository, https://github.com/andistoll/coincidence_detection_and_integration_behavior_in_SNNs
