# Supplementary material for: Coincidence detection and integration behavior in spiking neural networks
Source: Cogn Neurodyn. 2023 Dec 13;18(4):1753–65. doi: 10.1007/s11571-023-10038-0 (PMC11297875; doi:10.1007/s11571-023-10038-0)
Supplement: Supplementary file 1 — Supplementary file1 (PDF 1579 kb) [file 11571_2023_10038_MOESM1_ESM.pdf]

Supplemental Material

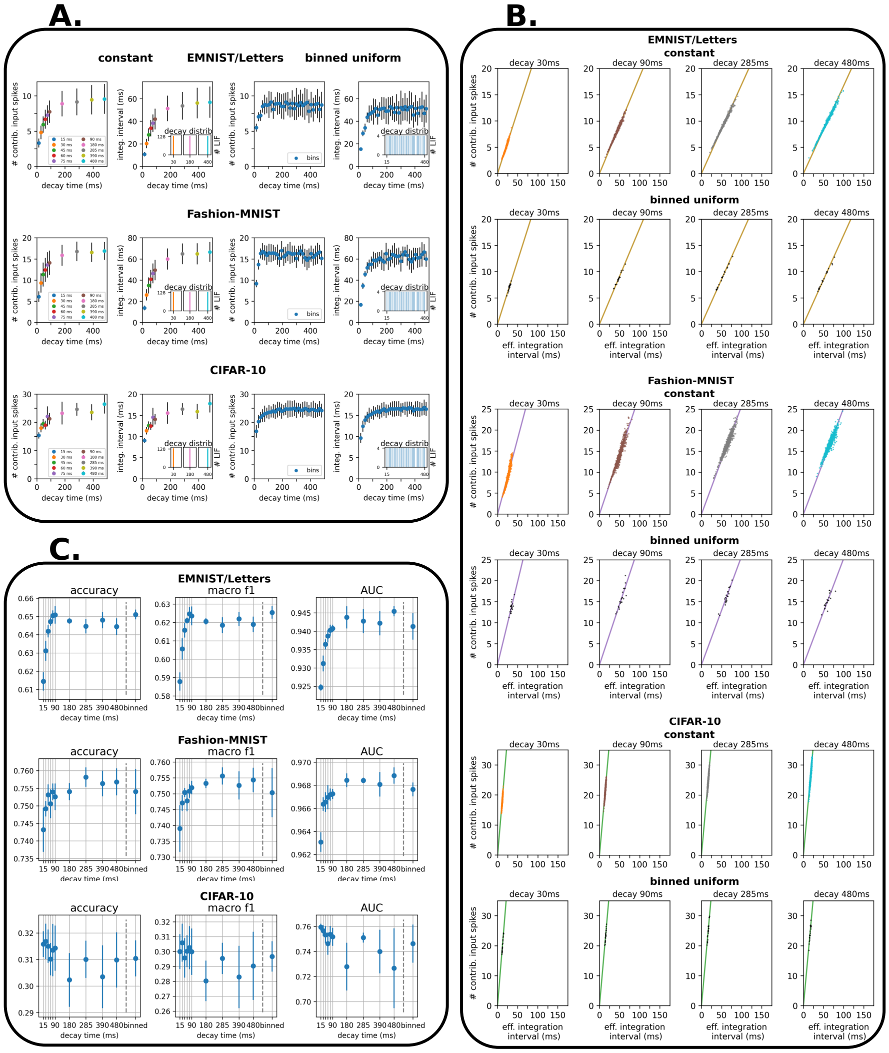

Figure 1: Complete analysis for datasets: EMNIST/Letters, Fashion-MNIST, CIFAR10; **A**: Contributing input spikes and effective integration interval as functions of the set decay time; **B**: Correlation of effective integration interval and contributing input spikes; **C**: Network performance for different decay times
